# Exploring the impact of seasonal variations on the chemical composition, antinociceptive, and anti-inflammatory properties of *Pogostemon heyneanus* Benth. essential oil

**DOI:** 10.3389/fphar.2024.1336878

**Published:** 2024-02-01

**Authors:** Paulo Vinicius Lima Santos, Lucas Botelho Jerônimo, Weyda Suyane Campos Ribeiro, Gustavo Moraes Lopes, João Henrique de Castro Leão Neto, Hugo Borges Oliveira da Silva, Pedro Iuri C. da Silva, Renata Cunha Silva, Joyce Kelly da Silva, Jofre Jacob S. Freitas, Rosa Helena V. Mourão, William N. Setzer, José Guilherme S. Maia, Pablo Luis B. Figueiredo

**Affiliations:** ^1^ Programa de Pós-graduação em Ciências Farmacêuticas, Instituto de Ciências da Saúde, Universidade Federal do Pará, Belém, Brazil; ^2^ Laboratório de Química dos Produtos Naturais, Universidade do Estado do Pará, Belém, Brazil; ^3^ Laboratório de Morfofisiologia Aplicada a Saúde, Departamento de Morfologia e Ciências Fisiológicas, Universidade do Estado do Pará, Belém, Brazil; ^4^ Programa de Pós-graduação em Farmacologia e Bioquímica, Instituto de Ciências Biológicas, Universidade Federal do Pará, Belém, Brazil; ^5^ Laboratório de Bioprospecção e Biologia Experimental, Universidade Federal do Oeste do Pará, Santarém, Brazil; ^6^ Aromatic Plant Research Center, Lehi, UT, United States

**Keywords:** oriza, volatile constituents, patchouli alcohol, analgesic activity, sesquiterpenes

## Abstract

**Background:** *Pogostemon heyneanus* leaves infusions are relevant in ethnopharmacology for treating colds, coughs, headaches, and asthma.

**Purpose:** The essential oil chemical composition of a *Pogostemon heyneanus* specimen was monthly monitored from October 2021 to July 2022 to evaluate the climatic influences on its yield and chemical composition and antinociceptive, andanti-inflammatory properties.

**Methods:** The leaves, collected monthly over a 10-month period, were submitted to hydrodistillation. The oils obtained were analyzed by gas chromatography coupled to a mass spectrometer and gas chromatography coupled to flame ionization detector. The *P. heyneanus* essential oil (PhEO) was tested *in vivo* to evaluate its peripheral analgesic actions through the abdominal writhing test induced by acetic acid, and peripheral analgesia by tail immersion. Neurogenic and inflammatory pain were evaluated by formalin test, and acute oral toxicity of the oil was also verified.

**Results:** PhEO presented 27 chemical constituents with the highest predominance of patchoulol (43.6%–76.9%), α-bulnesene (0.2%–12.7%), α-guaiene (0.4%–8.9%), seychellene (3.8%–5.1%) and pogostol (0.0%–8.2%). The climatic parameters insolation, humidity, rainfall, and temperature did not influence the essential oil yield or the main chemical constituents, except for pogostol, which presented a strong (r = 0.73) and statistically significant (*p* < 0.05) correlation with temperature. PhEO did not display toxicity at the maximum 300 mg/kg dosage. The oil showed low peripheral and central analgesic action at 100 mg/kg, while in the neurogenic and inflammatory pain inhibition tests, no actions related to PhEO were observed. In the carrageenan-induced peritonitis test, PhEO did not reduce the migration of leukocytes to the peritoneal cavity compared to the control group.

**Conclusion:** *Pogostemon heyneanus* is a resistant plant to seasonal influences and a source of patchoulol. Despite ethnopharmacological indications, no *in-vivo* biological activities such as neurogenic or inflammatory pain were identified in the present work. So, the low influence of the climatic parameters on chemical composition can infer that the low pharmacological activity is also not subject to climatic variations, that is, it does not change due to the climate.

## 1 Introduction

Among the species in the Amazon, *Pogostemon heyneanus* Benth. (Lamiaceae), commonly known as oriza, patchouli, or pathuli, is an herbaceous species native to Southeast Asia, widely distributed in India, Malaysia, and Indonesia (Lorence et al., 2020).

*Pogostemon heyneanus* essential oil is an additive known mainly for its use in cosmetology and aromatics (Cabral, 2020; Musta et al., 2021). Furthermore, infusions of oryza leaves are relevant in ethnopharmacology for treating colds, coughs, headaches, and asthma (Rabelo et al., 2022). Phytochemical studies with this essential oil demonstrated the occurrence of antioxidant (Rai et al., 2016), antifungal (Botelho et al., 2022), bactericidal against multidrug-resistant strain of methicillin-resistant *Staphylococcus aureus* (MRSA) (Elmesseri et al., 2022), and anti-inflammatory (Cabral, 2020), which can be attributed its chemical composition.

There are only a few studies on the chemical constituents of *P. heyneanus* essential oils. Maia and Andrade (2009) demonstrated that *P. heyneanus* essential oil from the Amazon had patchoulol (patchouli alcohol), α-guaiene, aromadendrene, and α-bulnesene as its main components. Another phytochemical study carried out with the volatile oil of this species, obtained in Italy, described the occurrence of α-bulnesene, seychelene, α-guaiene, and patchoulol (Souza Filho et al., 2009).

Moreover, many studies reported that the chemical composition and essential oils yield depend on age, circadian rhythm, soil, temperature conditions, plant organ, and extraction method, among others (Benomari et al., 2023). Therefore, studies are necessary to analyze the influence of these factors on the chemical variability of essential oils, knowledge of which is essential for applying essential oils for phytotherapeutic purposes.

The research for essential oils anti-inflammatory activities has increased during the last years (Miguel, 2010). Moreover, many essential oils contain active anti-inflammatory compounds, which mediate anti-inflammatory effects via different mechanisms. Thus, the treatment with essential oil can decrease the expression of pro-inflammatory cytokines and mediators, neutralize excess reactive oxygen species, alleviate tissue edema, and accelerate wound healing due to infection (Zhao et al., 2023).

Compared to conventional anti-inflammatory drugs and small molecules (which target a specific pathway and their known side effects), complex essential oil compositions can safely target multiple signaling pathways associated with inflammation. However, only a few clinical trials have been conducted to evaluate the anti-inflammatory effect of PEOs (Zhao et al., 2023).

Although *P. heyneanus* essential oil has *in-vitro* biological potential, few *in-vivo* studies are available in the literature on the chemical composition and analgesic and anti-inflammatory actions, such as the toxic activity of its essential oils. Therefore, this work aimed to evaluate the influence of seasonality on the chemical composition of *P. heyneanus* essential oil, its acute oral toxicity, and its antinociceptive and anti-inflammatory. The information gained from this study could have an impact as to when this medicinal plant is collected and the implications for treatment protocols and expectations.

## 2 Materials and methods

### 2.1 Plant material and essential oil extraction

The *P. heyneanus* Benth. Leaves were collected in Mosqueiro Island, city of Belém, Pará State (Latitude 1°5′58.095″S; longitude 48°24′1.530″W), the single specimen samples were obtained following Brazilian laws regarding the protection of biodiversity (SISGEN AED714B). For the seasonal study, fresh leaves (30 g) were collected on the second day of each month, at 10 a.m. from October 2021 to July 2022. After identification, a voucher (MG246329) was deposited in the Herbarium “João Murça Pires”, from the Museu Paraense Emílio Goeldi.

Leaves of *P. heyneanus* were allowed to dry for 7 days at room temperature. Hydrodistillation of the dry leaves was carried out using a glass Clevenger apparatus for 3 hours in triplicate; the condenser was maintained between 10°C and 15°C with a recirculating chiller. The essential oil was dehydrated by centrifugation (5 min, 3,000 rpm) with anhydrous Na_2_SO_4_ and repeated centrifugation. The percent yield of essential oil expressed in percent (v/w) and calculated from humidity-free biomass through the relationship between oil volume, mass plant sample, and humidity calculated as follows 100% × volume essential oil/mass dry leaves (Barros et al., 2022).

### 2.2 Climatic data

The climatic parameters of insolation, precipitation, relative humidity, and average temperature were obtained monthly in parallel to the collection periods on the website of the National Institute of Meteorology (http://www.inmet.gov.br/portal/, accessed on 24 April 2023) from the Brazilian Government (INMET, 2023). Meteorological data were recorded using the automatic station A-201, located in Belém, Pará state, Brazil, equipped with a Vaisala system, model MAWS 301 (Vaisala Corporation, Helsinki, Finland).

### 2.3 GC and GC-MS analyses

Gas chromatography coupled to mass spectrometer (GC-MS) and gas chromatography with flame ionization detector (GC-FID) were used to analyze the composition of *P. heyneanus* essential oils. A Shimadzu Model QP 2010 ultra instrument (Shimadzu, Tokyo, Japan) equipped with Rtx-5MS fused silica capillary column (30 m, 0.25 mm; 0.25 μm film thickness) as stationary phase was used (Restek, Bellefonte, PA, United States). Helium gas was adjusted to 1.0 mL/min at 57.5 kPa as carrier gas. The injection of diluted essential oils in hexane (1µL, 5 μL of each oil: 500 μL of *n*-hexane) was injected in the split mode (1:20). The injector and interface temperatures were 250°C; The programmed oven temperature was 60°C–240°C (3°C/min), followed by a 10 min isotherm. Electron ionization mass spectrometry (EIMS) at 70 eV, the ion source temperature was 200°C.

The mass spectra were obtained using automatic scanning, with fragment mass in the 35–400 m/z range. The comparison of mass spectra and retention indices presented by the samples was made using the similarities between the mass spectra and retention indices presented in the commercial libraries FFNSC-2 (Mondello, 2011) and Adams (2007). The retention indices of volatile constituents were calculated using the linear equation of Van Den Dool and Kratz (1963), using a homologous series of hydrocarbons (C_8_–C_40_, Sigma-Aldrich, St. Louis, MO, United States) under the same chromatographic conditions.

GC-FID analysis was performed for each essential oil sample on a Shimadzu QP-2010 instrument (Shimadzu, Tokyo, Japan) equipped with an FID detector under the same conditions described above, except hydrogen was used as the carrier gas. The percentage composition of the oil sample was calculated from the GC-FID peak areas. Analyses were carried out in triplicate (i.e., three different essential oil samples injected separately).

### 2.4 Animals and ethical statement

Adult male mice (*Mus musculus*, 30–40 g, Swiss) were obtained from the Evandro Chagas Institute and retained in the “Luiz Carlos de Lima Silveira” animal facility of the Universidade do Estado do Pará (UEPA). The mice were maintained under standard conditions of 12-h light/dark cycle, temperature 22°C–25°C, humidity 65%, food and water available *ad libitum*. The research was approved by the UEPA Animal Use Ethics Committee (CEUA) under protocol 04/2023.

### 2.5 Acute oral toxicity

Acute oral toxicity was assessed according to Organization for Economic Co-operation and Development (OECD) guidelines 423 (OECD, 2002). The animals were randomly distributed into two groups. The first group was treated with distilled water, and the second group was treated with *P. heyneanus* essential oil (PhEO) at 300 mg/kg. Treatments were carried out by gavage (10 mL/kg). After the treatments, the animals were observed for 2 h and then every 24 h for 14 consecutive days. During this period, behavioral and physiological changes were observed (alertness, spontaneous motor activity, locomotion, apathy, response to touch, stereotypy, aggression, ataxia, sweating, urination, diarrhea, convulsions, and death). On the 15th day, the animals were subjected to induced euthanasia (ketamine 300 mg/kg and xylazine 30 mg/kg) (Souzados et al., 2021).

Blood was obtained by cardiac puncture in order to analyze renal (creatine and urea) and liver [alanine aminotransferase (ALT-PGP), aspartate transaminase (AST-TGO) and alkaline phosphatase (ALP)] function. Analyses were carried out using an Olympus AU 400 Autoanalyzer (Hamburg, Germany) (Costa-Silva et al., 2008).

### 2.6 Analysis of analgesic activity

#### 2.6.1 Peripheral analgesic effect in the abdominal contortion test

The acetic acid-induced abdominal contortion assay was used to assess the peripheral analgesic potential (Koster, 1959; Conegundes et al., 2021). The mice were randomly divided into five groups of five mice: Control group (treated with 0.9% saline solution, p.o.), three treatment groups (PhEO doses of 50, 100, and 300 mg/kg, p. o.), and a positive control group (5 mg/kg indomethacin, p.o.). Sixty minutes after treatment, the mice were subjected to 10 mL/kg of 0.6% acetic acid i.p. After administration of the acetic acid, the animals were confined for 30 min and the abdominal contortions and hind paw extensions were recorded. The analgesic effect was assessed based on the reduction in the number of abdominal contortions.

#### 2.6.2 Tail immersion test

The tail immersion test evaluates the central analgesic activity by submerging the animals’ tail in high temperatures. This test evaluates the latency time (s), defined as the period for the animal to remove its tail. Before the start of the test, the animals were subjected to a pre-test, and those with latency values greater than 5 s were excluded. The selected animals were separated into groups (n = 6) that were treated with vehicle (1% Tween 80 diluted in sterile saline solution, p. o.), morphine administered at 5 mg/kg (i.p.) or PhEO at doses of 100 and 300 mg/kg (p.o.). Subsequently, ⅓ (approximately 2 cm) of the animals’ tails were submerged in the liquid with a fixed temperature of 55ºC ± 0.5°C, and the complete tail withdrawal response was evaluated at times 0, 30, 60, 90, and 120 min (Hamura et al., 2000; Gammoh et al., 2023).

#### 2.6.3 Formalin test

The mice were separated into four groups: Control group (1% Tween 80 in distilled water, 10 mL/kg, p.o.), treatment groups (PhEO at doses of 100, and 300 mg/kg, p.o.), and positive control (morphine, 4 mg/kg, i.p.). After 1 h, the animals received 20 µL of formalin (1.0%) in the right hind paw (subplantar area) and were then placed individually in plastic containers. The time (seconds) during which the animal licked the injected paw was recorded during the first phase (0–5 min), attributed to the neurogenic phase, and the second phase (15–30 min) was characterized as inflammatory pain (Hunskaar and Hole, 1987; Gomes et al., 2007; Liu et al., 2021).

### 2.7 Analysis of anti-inflammatory activity

#### 2.7.1 Carrageenan-induced peritonitis test

The carrageenan-induced peritonitis assay was used to assess cell migration during the inflammatory process (Souza and Ferreira, 1985). The mice were divided into five groups of six mice: Control group (no treatment), saline group (0.9% saline solution, i.p.), treatment groups (PhEO at doses of 100 and 3,000 mg/kg, i.p.), and positive control group (dexamethasone 1 mg/kg, i.p.). After 1 h, the control group received saline and the other groups received carrageenan (300 μg/mL in sterile saline), i.p. Four hours after injection, the mice were euthanized and their peritoneal cavities were rinsed with 3 mL of ice-cold phosphate-buffered saline (PBS) containing ethylenediaminetetraacetic acid (EDTA, 3 μM). Total leukocytes in the peritoneal fluid were determined by pipetting 20 µL of peritoneal fluid into 400 µL of Turk’s solution. An aliquot was read in a Neubauer chamber (Kasvi, Brazil) using a Nikon Eclipse E100 binocular microscope (Nikon Europe BV).

### 2.8 Statistical analysis of chemical composition and *in vivo* test

Statistical analysis was evaluated using the Tukey test (*p* < 0.05), and Pearson correlation coefficients (r) were calculated to determine the relationship between the analyzed parameters such as insolation, precipitation, relative air humidity, and average temperature), using the GraphPad Prism software method, version 8.0. Principal component analysis (PCA) was applied to check the interrelationship in essential oil components (>1.0%) (OriginPro trial version, OriginLab Corporation, Northampton, MA, United States). Hierarchical cluster analysis (HCA), considering Euclidean distance and Ward linkage, was used to check the similarity of oil samples based on the distribution of constituents selected in the previous PCA analysis (da Cruz et al., 2022).

Statistical evaluations of the *in-vivo* experimental data were carried out using GraphPad Prism version 5.0. The data are expressed as means ± standard errors. Statistically relevant differences were determined using the ANOVA test, followed by Tukey’s *post hoc* test. Differences were deemed to be statistically significant between treatment groups when *p* < 0.05 (de Jesus et al., 2023).

## 3 Results

### 3.1 Seasonal effects on oil yield

**FIGURE 1:**
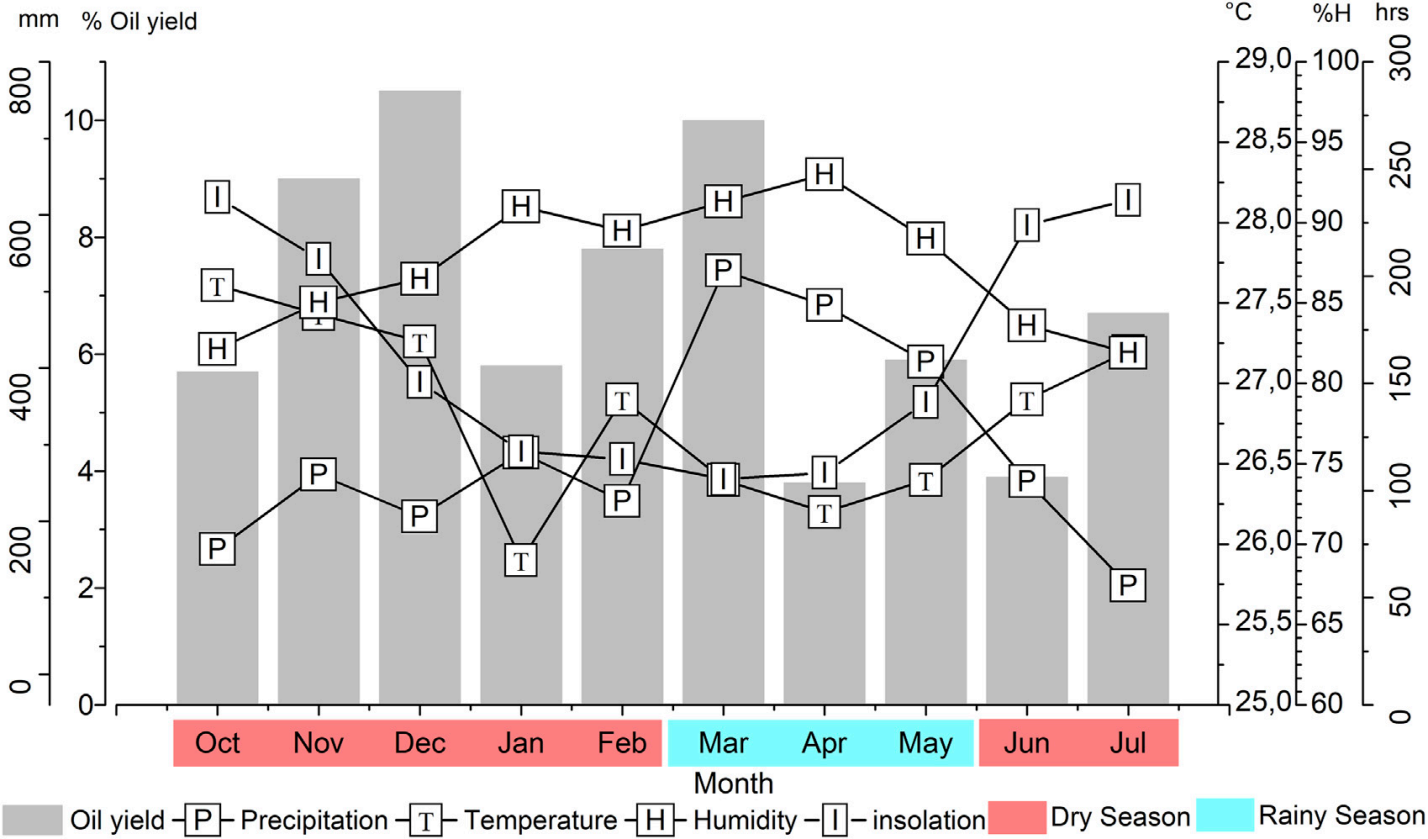
Relationship between climatic parameters and oil yield of *Pogostemon heyneanus* during the seasonal study.

*Pogostemon heyneanus* essential oil yields varied from 3.8% (April) to 10.5% (December), with an average of 6.9% ± 2.4% during the studied period (Figure 1). Essential oil production did not show a significant difference (*p* < 0.05) during the period of lowest rainfall intensity (7.1% ± 2.2%) and highest intensity (6.6% ± 3.2%) in the Tukey test. Regarding climatic parameters with essential oil content, no significant correlation was observed with climatic parameters (temperature, humidity, insolation, and precipitation) (*p* > 0.05).

### 3.2 Seasonal effects on oil chemical composition

Table 1 presents the 27 chemical constituents identified by GC and GC-MS in the essential oils of *P. heyneanus* leaves, which are listed in ascending order of their respective retention indexes (RI). The compounds comprise about 88.9% of the total volatile constituents in the oils during the seasonal study. The predominant classes in leaf samples were oxygenated sesquiterpenes (54.3%–86.5%) followed by hydrocarbon sesquiterpenes (9.2%–35.9%).

**TABLE 1:** Constituents identified in *Pogostemon heyneanus* essential oils during the seasonal study.

IRC	IRL	Oil yield constituents	Oct	Nov	Dec	Jan	Feb	Mar	Apr	May	Jun	Jul	Mix
			5.7	9.0	10.5	5.8	7.8	10.0	3.8	5.9	3.9	6.7	
			(%)										
932^a^	934	α-pinene		tr						0.1			
969^a^	973	sabinene						tr	tr	**0.1**	0.6	0.3	
974^a^	978	β-pinene		tr				tr		tr	0.1	0.1	
1024^a^	1,029	limonene		tr						tr			
1335^a^	1,339	δ-elemene	tr	tr	tr		0.5		tr	0.1		0.1	0.2
1379^a^	1,383	β-patchoulene	0.3	0.6	0.3	0.7		0.7	0.9	1.1	tr	1.2	2.5
1389^a^	1,394	β-elemene	0.7	0.7	0.7	0.8		0.7	0.7	0.6	0.6	1.2	1.3
1406^a^	1,411	cycloseychellene	0.3	0.3	0.3	0.2	0.2	0.3	0.2	0.3	0.4	0.4	0.7
1417^a^	1,421	*E-c*aryophyllene	0.6	1.2	0.6	1.6		1.5	1.5	2.2	0.1	2.3	2.1
**1437** ^**a**^	**1,441**	**α-guaiene**	**1.9**	**3.8**	**2.0**	**5.8**		**5.2**	**5.4**	**8.9**	0.4	**8.2**	**7.2**
**1444** ^**a**^	**1,444**	**seychellene**	**4.3**	**4.4**	**4.5**	**3.1**	**4.7**	**4.8**	**3.8**	**4.0**	**5.1**	**4.5**	**5.2**
1452^a^	1,455	α-humulene	0.3	0.5	0.3	0.5			0.5	0.6		0.6	1.2
1454^a^	1,458	α-patchoulene	1.8	2.2	1.8	1.9	0.9	2.6	2.1	2.9	1.4	3.0	4.2
1464^a^	1,462	9*-epi-E*-caryophyllene		1.0	0.8			0.4	0.6	2.1	0.7	1.1	
1489^a^	1,491	β-selinene	0.1	0.1	tr			0.1	0.1	0.3		0.2	0.2
1489^a^	1,494	α-selinene	0.1		0.1					0.1			
1501^a^	1,501	aciphyllene	0.4	0.8	0.4	1.4		1.0	1.1	2.3	0.1	1.9	3.1
**1509** ^**a**^	**1,508**	**α-bulnesene**	**3.0**	**5.8**	**3.2**	**8.8**	0.2	**8.0**	**7.7**	**12.7**	0.6	**11.1**	**8.6**
1520^a^	1,520	7-*epi*-α-selinene	0.1	0.1	0.1			0.1	0.1	0.2		0.1	0.3
1548^a^	1,551	elemol	0.1	0.1	0.1	0.1		0.1	0.1	0.1	0.1	0.1	0.1
1553^a^	1,558	norpatchoulenol	1.2	0.9	0.8	1.1	0.8	0.8	1.2	0.9	1.5	0.9	1.8
1576^b^	1,576	spathulenol			1.5		1.8	0.6	0.3		1.9	0.4	
1587^a^	1,585	caryophyllene oxide	2.0	1.3	2.1	0.1	2.2	1.0	0.6	0.3	2.9	0.6	
1608^a^	1,612	humulene epoxide II	0.6		0.5						0.8		
1630^a^	1,631	muurola-4,10 (14)-dien-1β-ol							0.2			0.2	
**1651** ^**a**^	**1,651**	**pogostol**	0.4		0.4	**6.0**	**5.6**	**5.6**	**6.5**	**8.2**	**6.6**	**6.3**	**1.0**
**1668** ^**b**^	**1,669**	**patchoulol**	**68.6**	**63.2**	**66.1**	**62.2**	**76.9**	**56.5**	**59.5**	**43.6**	**61.2**	**46.7**	**60.4**
		monoterpene hydrocarbons	0.0	0.0	0.0	0.0	0.0	0.0	0.0	0.2	0.7	0.4	0.0
		sesquiterpene hydrocarbons	13.8	21.4	15.0	24.8	6.5	25.3	24.6	38.1	9.2	35.9	36.8
		oxygenated sesquiterpenoids	71.6	64.6	70.7	68.5	86.5	63.8	67.1	52.1	73.5	54.3	61.5
		total identified	85.4	86.0	85.7	93.3	93.0	89.1	91.7	90.4	83.3	90.6	98.3

RIC, calculated retention index; RIL, literature retention index; a, Adams (2007); b, Mondello (2011); Main constituents in bold; Standard deviation was less than 2.0 (n = 3).

**FIGURE 2:**
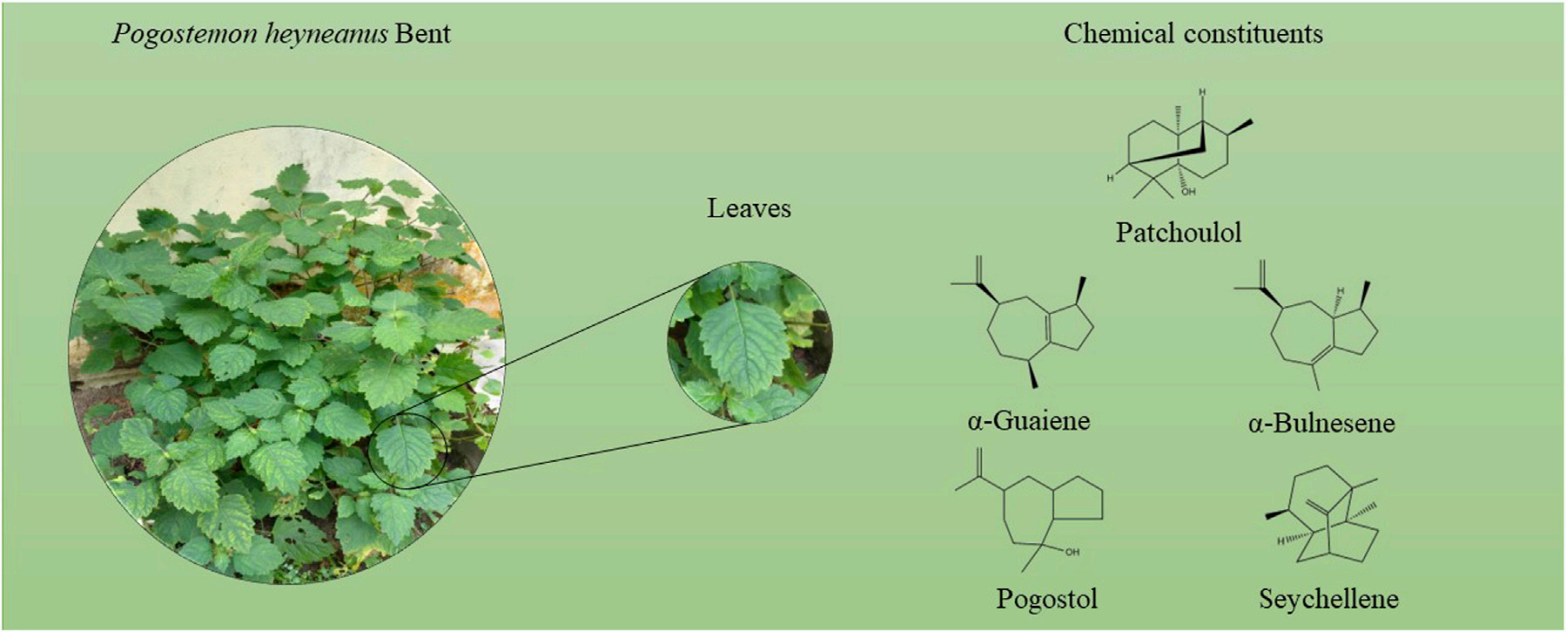
Chemical constituents identified in the essential oils of *Pogostemon heyneanus*.

The main chemical constituents (>5%) identified in oils from the seasonal study were patchoulol (patchouli alcohol, PA) (43.6%–76.9%), α-bulnesene (0.2%–12.7%), α-guaiene (0.4%–8.9%), seychellene (3.8%–5.1%), and pogostol (0.0%–8.2%) as shown in Figure 2 below.

The constituents patchoulol, α-bulnesene, α-guaiene and seychellene and their respective classes did not significantly correlate with seasonal parameters. However, the pogostol constituent showed a negative and statistically significant correlation (*p* < 0.05) with temperature (r = −0.73), and the monoterpene hydrocarbons class showed a negative very strong correlation with temperature (r = −0.92), strong correlation with insolation (r = −0.62) and weak correlation with precipitation (r = −0.47), as shown in Table 2 below.

**TABLE 2:** Correlation between yield, main components, and classes of compounds of *Pogostemon heyneanus* essential oil and climatic parameters.

Parameter	Temperature	Humidity	Insolation	Precipitation
oil yield	0.30	0.02	−0.18	−0.03
β-patchoulene	−0.34	0.16	−0,07	0.28
β-elemene	0.04	−0.31	0.39	−0.14
*E*-caryophyllene	−0.38	0.17	−0.09	0.30
α-guaiene	−0.42	0.20	−0.12	0.32
seychellene	0.58	−0.47	0.38	−0.19
α-patchoulene	−0.13	−0.04	0.10	0.27
9-*epi*-*E*-caryophyllene	−0.01	−0.10	0.13	0.16
aciphyllene	−0.43	0.17	−0.11	0.26
α-bulnesene	−0.45	0.24	−0.16	0.36
norpatchoulenol	−0.02	−0.28	0.44	−0.11
spathulenol	0.16	−0.08	−0.05	−0.20
caryophyllene oxide	0.61	−0.47	0.38	−0.42
pogostol	−0.73^a^	0.43	−0.37	0.43
patchoulol	0.28	0.03	−0.10	−0.28
monoterpene hydrocarbons	−0.92^a^	−0.12^a^	−0.62^a^	−0.47^a^
sesquiterpene hydrocarbons	−0.10	−0.12	−0.05	−0.26
oxygenated sesquiterpenes	0.18	0.22	0.09	0.37

^a^ Significant correlation (*p* < 0.05).

### 3.3 Multivariate analysis of *Pogostemon heyneanus*

The chemical variability of oil samples of *P. heyneanus* was evaluated by multivariate statistical analyses (HCA, hierarchical cluster analysis; PCA, principal component analysis) using constituents with levels greater than 1.0%.

**FIGURE 3:**
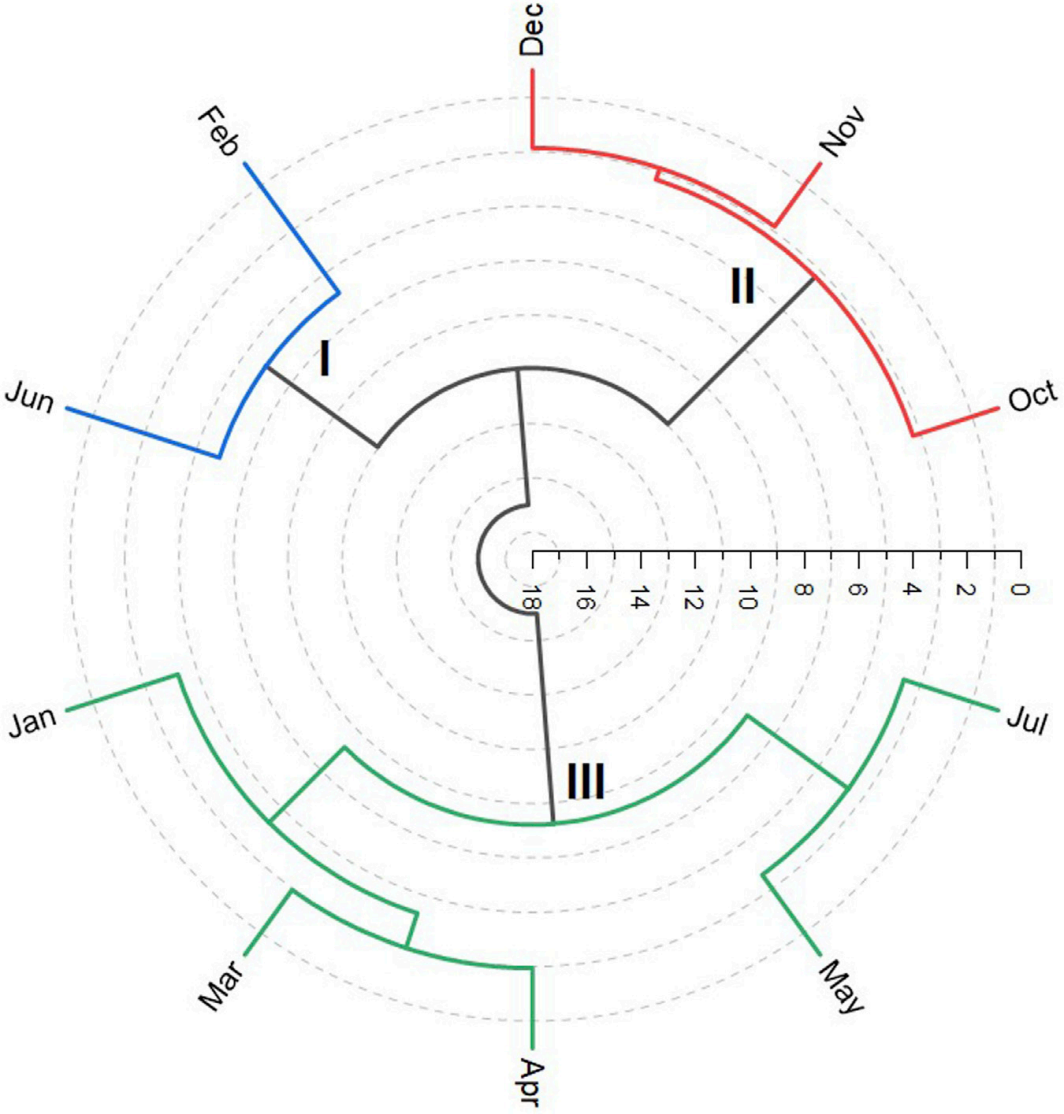
Hierarchical cluster analysis of the monthly *Pogostemon heyneanus* essential oils collection.

Applying hierarchical cluster analysis (HCA) provided the dendrogram presented in Figure 3; the analyzed volatiles’ compositions were included in three groups. Group I is comprised of oils from leaves collected in February and June. Group II includes oil samples from October, November, and December; Group III includes samples from January, March, April, May, and July.

**FIGURE 4:**
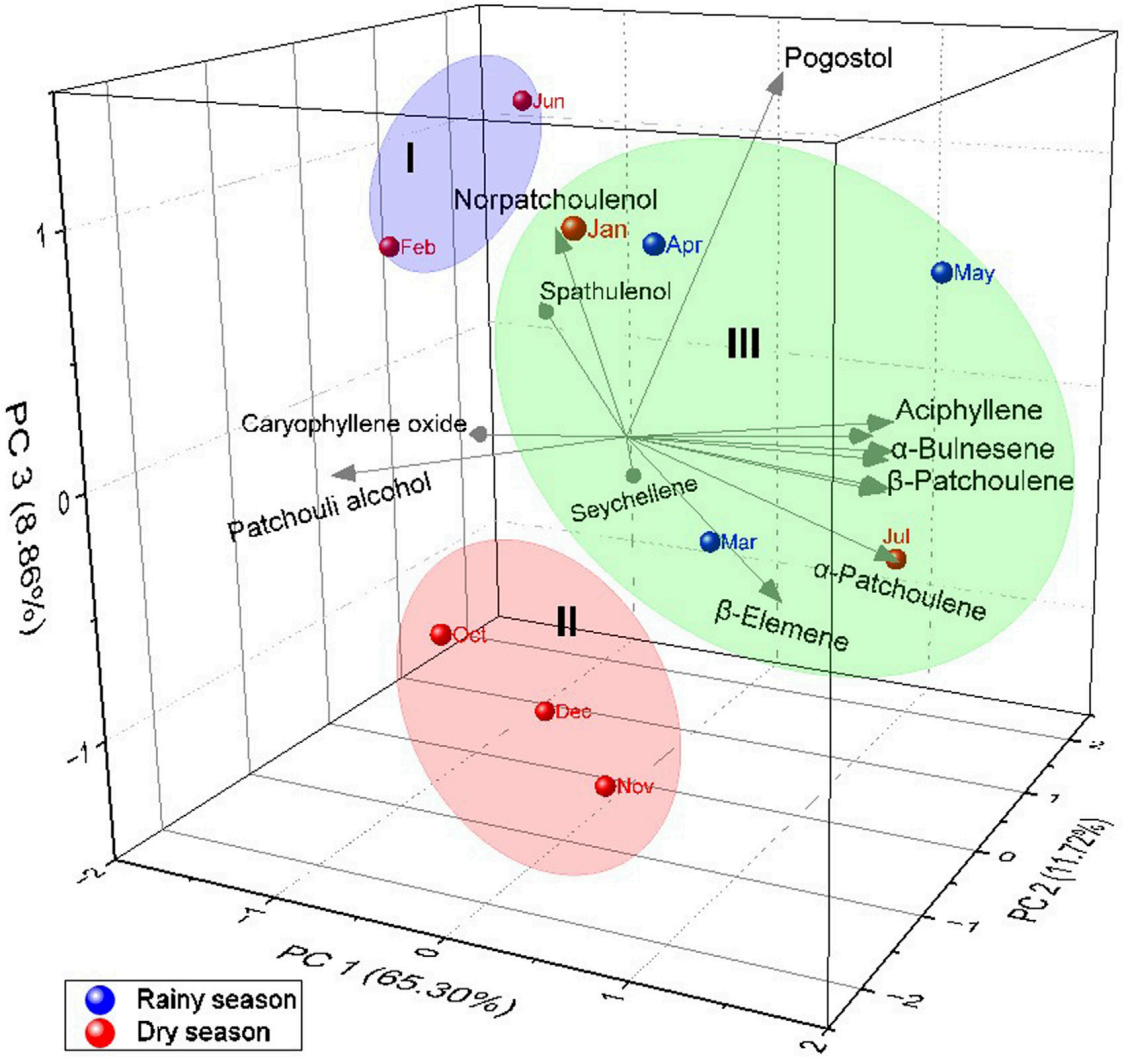
Principal components Analysis of main components in *Pogostemon heyneanus* essential oil.

Principal Component Analysis (PCA, Figure 4) elucidated 85.88% of the data variability. PC1 explained 60.30% and showed negative correlations with seychellene (r = −0.73), norpatchoulenol (r = −0.32), spathulenol (r = −1.10), caryophyllene oxide (r = −1.36) and patchoulol (r = −1.33). The second component PC2 explained 11.72%, showed negative correlations with β-patchoulene (r = −0.05), β-elemene (r = −0.21), *E*-caryophyllene (r = −0.03), α-bulnesene (r = −0.03) and patchoulol (r = −1.03). The third component PC3 explained 8.86% of the data and showed positive correlations with α-guaiene (r = 0.06), α-bulnesene (r = 0.04), norpatchoulenol (r = 0.85), spathulenol (r = 0.30) and pogostol (r = 1.41). Like the HCA, the PCA analysis confirmed the formation of three distinct groups.

**FIGURE 5:**
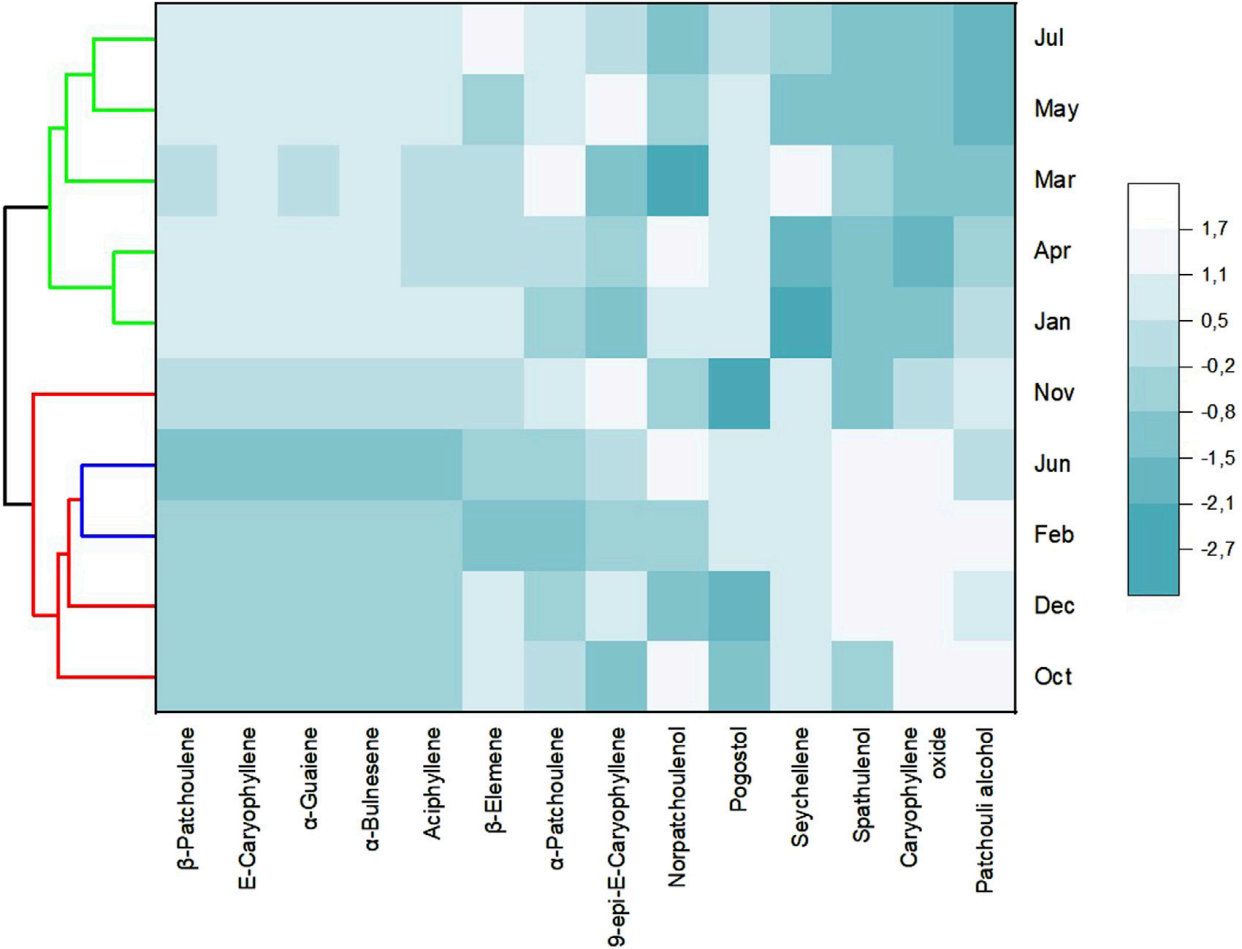
Heat map of class clustering in essential oils from *Pogostemon heyneanus* samples.

Applying additional multivariate analysis, in the heat map analysis combined with the hierarchical cluster analysis (Figure 5), with the classes of compounds, the color pattern that varied with the intensity of the color increased, indicating from the lowest to the highest degree. The clustered heatmap confirmed the clustering results obtained in PCA and HCA. However, correlations were observed between climatic parameters and oil constituents and their compound classes, as mentioned previously (see Table 2).

### 3.4 Acute oral toxicity

After 14 days of analysis, no signs of mortality were observed, and there was little change in the behavioral patterns of the animals treated with the 300 mg/kg of PhEO. Furthermore, there were no observed differences in the relative organ masses between the PhEO treatment groups and the control group (data not shown).

There were no differences in biochemical parameters related to renal function (creatinine and urea) between the group treated with PhEO and the control. The biochemical parameters for evaluating liver function (alanine aminotransferase (ALT), aspartate aminotransferase (AST) and alkaline phosphatase (ALP)), only AST and ALP, were statistically significant compared to control (Table 3). However, these differences are within the reference values ​​for the species used in the study (Silva-Santana et al., 2020).

**TABLE 3:** Blood biochemical parameters of mice treated with *Pogostemon heyneanus* essential oil and the control group.

Parameters	Group control	PhOE group 300 mg/kg
ALT (U/L)	56.17 ± 13.95	55.60 ± 9.91 ^ns^
AST (U/L)	126.67 ± 53.71	80.40 ± 15.40^**^
ALP (U/L)	373.57 ± 65.41	213.00 ± 19.07^**^
Urea (mg/dL)	67.00 ± 10.17	66.40 ± 4.34 ^ns^
Creatinine (mg/dL)	0.18 ± 0.09	0.19 ± 0.03 ^ns^

ALT, alanine aminotransferase; AST, aspartate aminotransferase; Alkaline phosphatase (ALP). Statistical differences were observed using ANOVA, followed by Tukey’s post-test. Each column represents the mean ± SEM, of 5 animals per group. *p < 0.05 and **p < 0.001 when compared to the control group. ns, No statistical difference.

### 3.5 Peripheral analgesic effect of PhEO in the acetic acid-induced abdominal writhing test

**FIGURE 6:**
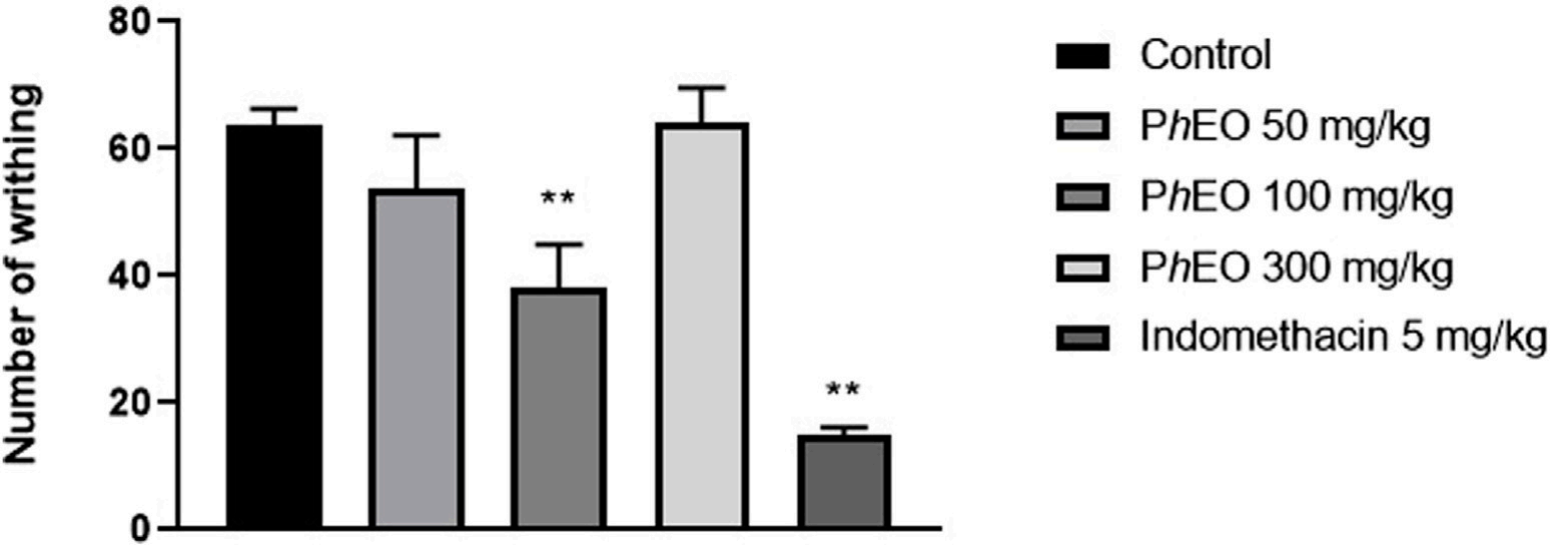
Analgesic effect of *Pogostemon heyneanus* essential oil (*Ph*EO, at 50, 100 and 300 mg/kg) in the abdominal writhing test induced by acetic acid (0.6% in saline, v/v) in mice. Each column represents the mean ± SEM (standard error of the mean) of 6 animals. **p < 0.01 was considered statistically significant compared to the control (ANOVA, followed by Tukey’s post-test).

In the current report, it was possible to verify, in Figure 6, a 41% contortions reduction in the group that received the dose of 100 mg/kg of PhEO compared to the control group (*p* < 0.01). No reduction in writhing was observed in the other groups treated with PhEO (50 and 300 mg/kg). Therefore, in the other antinociception tests, only 100, and 300 mg/kg doses were adopted. In animals treated with the standard drug (indomethacin 5 mg/kg), it was observed that the percentage of writhing inhibition was 77% (*p* < 0.01).

### 3.6 Central analgesic effect of PhEO in the tail immersion test

**FIGURE 7:**
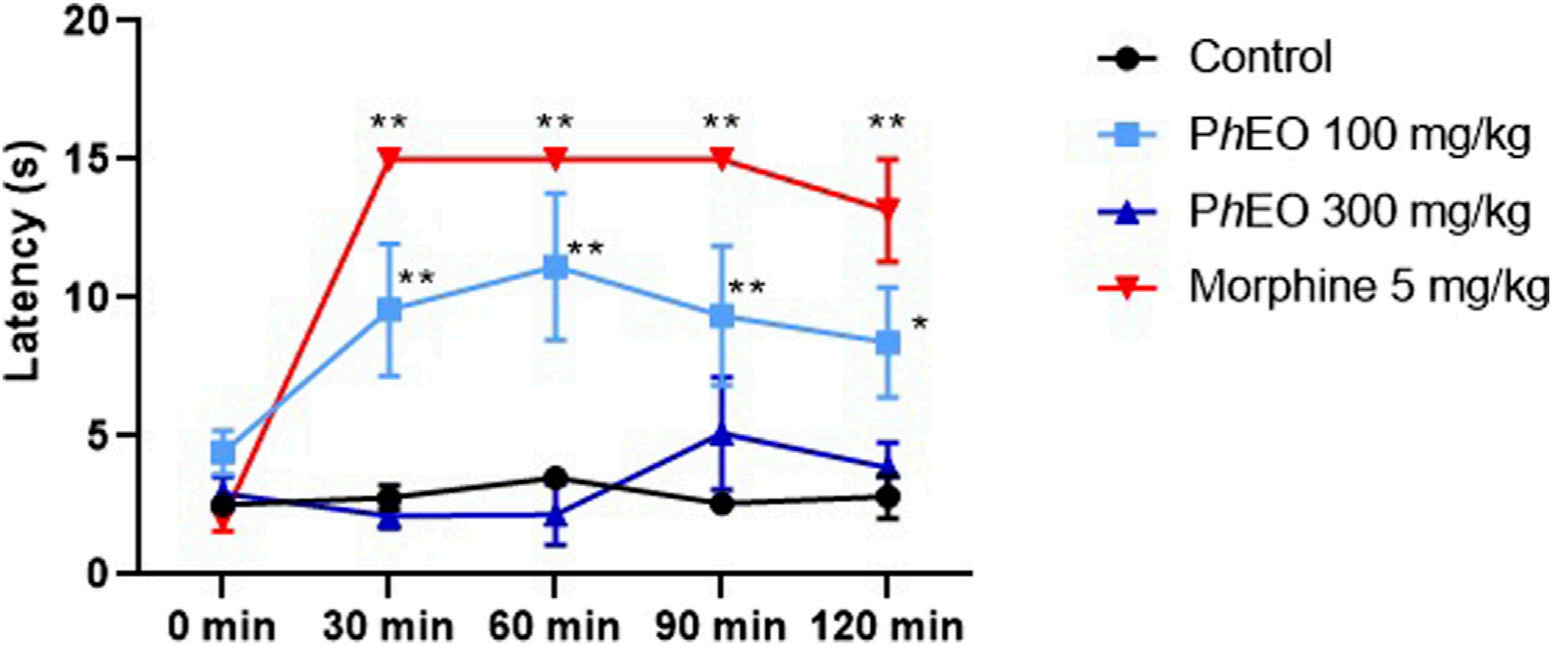
Central analgesic effect of *Pogostemon heyneanus* essential oil (*Ph*EO, at 100 and 300 mg/kg) in the tail dip test in mice. Each column represents the mean ± SEM (standard error of the mean) of 6 animals. **p < 0.01 was considered statistically significant compared to the control (ANOVA, followed by Tukey’s post-test).

The PhEO was only effective at a dose of 100 mg/kg, at times of 30 min (12.6 ± 4.04 s), 60 min (13.5 ± 3 s), 90 min (12.6 ± 4 s), and 120 min (11.5 ± 4.94 s), which showed longer latency times when compared to the control group 30 min (2.6 ± 1.15 s), 60 min (4 ± 0.89 s), 90 min (2.4 ± 0.89 s) and 120 min (2.6 ± 1.81 s). The morphine dose administered at 5 mg/kg showed a longer latency time compared to the control group in the periods 30 min (15 ± 0 s), 60 min (15 ± 0 s), 90 min (15 ± 0 s), and 120 min (13.2 ± 4.02 s). Therefore, a relevant action of PhEO on the central analgesic activity of mice is noticeable. The results are shown in Figure 7.

### 3.7 Effects of PhEO on the formalin test

**FIGURE 8:**
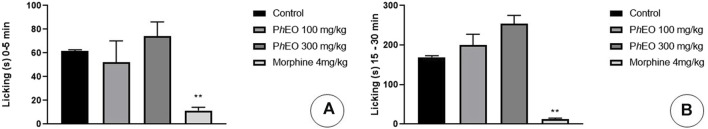
Central and peripheral analgesic effect of *Pogostemon heyneanus* essential oil (*Ph*EO, at 100 and 300 mg/kg) in the formalin test in mice. **(A)** first phase, assesses neurogenic pain; **(B)** second phase, assesses inflammatory pain. Each column represents the mean ± SEM (standard error of the mean) of 6 animals. **p < 0.01 was considered statistically significant when compared to the control (ANOVA, followed by Tukey’s post-test).

The results of the formalin test, both the first phase (neurogenic pain) and the second phase (inflammatory pain), are shown in Figure 8. Neither the PhEO dose of 100 mg/kg nor the dose of 300 mg/kg significantly decreased the licking time compared to the control group (*p* > 0.05). However, morphine, at 4 mg/kg, did significantly decrease licking time in both the first phase (81.67%, *p* < 0.01) and the second phase (92.46%, *p* < 0.01) compared to the control group.

### 3.8 Effects of PhEO on carrageenan-induced peritonitis test

**FIGURE 9:**
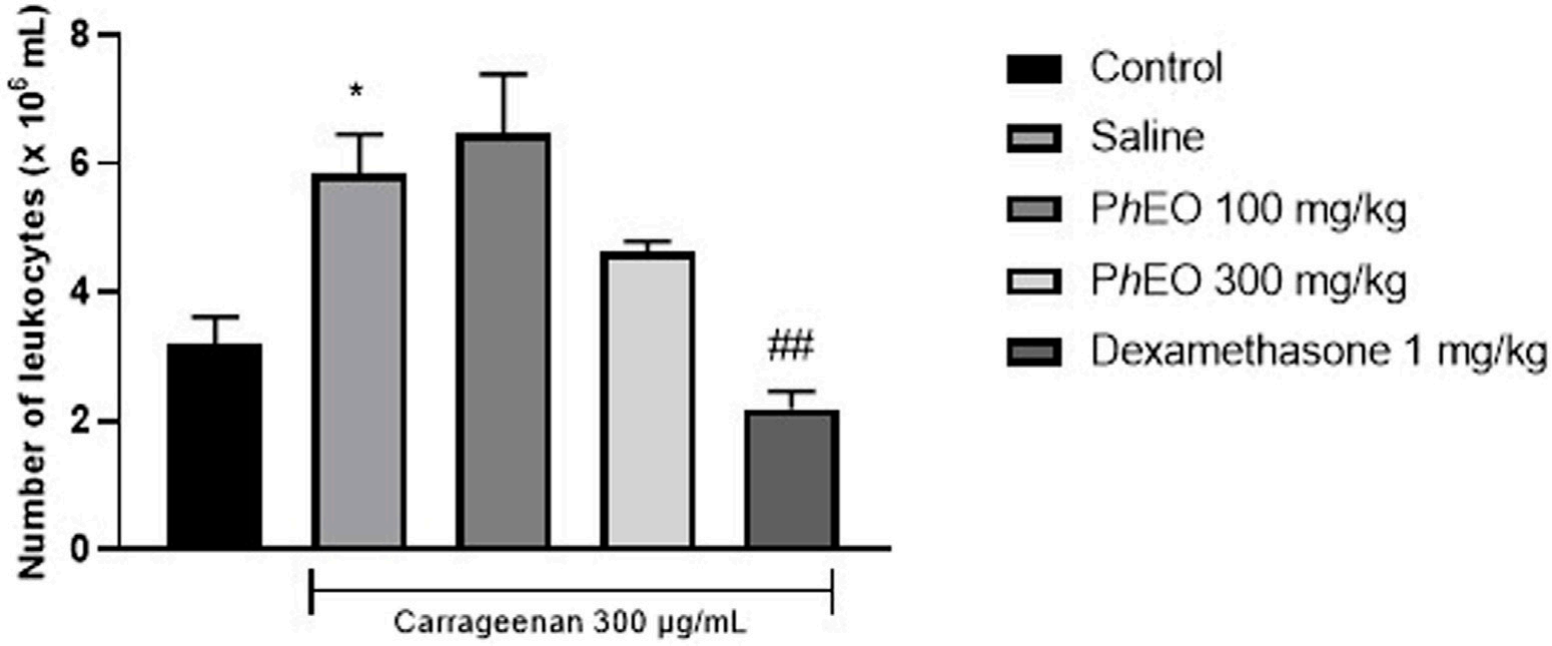
Effect of *Pogostemon heyneanus* essential oil (*Ph*EO, at 100 and 300 mg/kg) on carrageenan-induced peritonitis test. Each column represents the mean ± SEM (standard error of the mean) of 6 animals. *p < 0.05 compared to the control group. ##*p* < 0.01 compared to the saline group. (ANOVA, followed by Tukey’s post-test).

The total number of leukocytes results are presented in Figure 9. Carrageenan (saline group) was able to cause an increase in leukocyte migration (5.85 ± 1.34 leukocytes × 10^6^ mL) when compared to the control group (3.17 ± 0.97 leukocytes × 10^6^ mL), *p* < 0.05. PhEO, at the studied doses of 100 mg/kg (6.475 ± 2.24 leukocytes × 10^6^ mL) and 300 mg/kg (4.63 ± 0.38 leukocytes × 10^6^ mL) did not decrease the migration of leukocytes to the peritoneal cavity when compared to the saline group. However, dexamethasone (1 mg/kg) was able to reduce leukocyte migration (2.18 ± 0.60 leukocytes × 10^6^ mL) when compared to the saline group (*p* < 0.01).

## 4 Discussion

The Brazilian Amazon presents a precipitation regime modulated by dynamic atmospheric systems on micro, meso, and large scales. The Amazon region is characterized by only two seasons: dry and rainy. With the humid and hot climate, the Amazon has the highest rainfall from December to April, the rainy season, with the lowest rainfall from June to November in the dry season, and the other months are considered periods of transition between the seasons (Loureiro et al., 2014; da Costa et al., 2020). In a study that evaluated the effect of seasonality and essential oil, the climatic parameters obtained in 2022 showed an atypical climate in the year studied, with a period of greater rainfall intensity of just 3 months (March to May) (Santos et al., 2022).

During the study, patchoulol (PA) was the constituent with the highest oil content from *P. heyneanus* leaves. PA is a tricyclic oxygenated sesquiterpene and has several important pharmacological properties, such as immunomodulatory, anti-inflammatory, antioxidant, antitumor, antimicrobial, insecticidal, antiatherogenic, antiemetic, whitening, and sedative activities (Li et al., 2011; Hu et al., 2017).

PCA and HCA analyses did not differentiate *P. heyneanus* oil samples during the dry and rainy seasons. A previous study on the seasonality of essential oils from *Psidium friedrichsthalianum* leaves from Brazil showed no separation of samples from the dry and rainy seasons (Santos et al., 2023). Some species present variations in the content of constituents but cannot be separated in chemometric analyses due to their metabolism not correlating with biotic, abiotic, or climatic parameters, which can interfere with metabolic pathways (Santos et al., 2022; Guimarães et al., 2023). The results demonstrate that the essential oil was not toxic to animals. Based on the OECD guidelines (OECD, 2002), PhEO, with no acute deaths at 300 mg/kg, can be considered to have low acute oral toxicity. The intraperitoneal injection of acetic acid causes the release of inflammatory mediators such as prostaglandins, histamine, and several pro-inflammatory cytokines, which results in pain sensation (abdominal writhing) in the test animals (Dantas et al., 2020; Lee and Neumeister, 2020). The tail immersion test is an important evaluator of central analgesic activity in mice when studying the potential antinociceptive activity of a drug (Li et al., 2021). When the drug is effective, its activity in the nociceptive response determines a longer latency time in the animal compared to the control group, indicating a decrease in the activation of central nociceptors.

The formalin test is one of the most used models for selecting antinociceptive compounds. It consists of injecting formalin into the mouse’s hind paw, which produces nociception, which occurs in two phases (Gomes et al., 2007). The first phase, which assesses neurogenic pain, occurs in the 0–5 min range, and sees the activation of nociceptors, and primary afferent fibers, which release bradykinin and substance P. In the second phase, which analyzes inflammatory pain, occurs between 15 and 30 min, and causes the release of histamine, prostaglandins, serotonin, and bradykinin due to peripheral and spinal sensitization. This test is used because it is the one that most resembles clinical pain. Therefore, it was noticed that *P. heyneanus* essential oil does not reduce neurogenic pain and/or pain caused by inflammation (Dantas et al., 2020).

Carrageenan has been used for decades to induce inflammation in different animal models as a screening method in research to assess potential anti-inflammatory substances. Injection of carrageenan into the peritoneal cavity induces several pro-inflammatory mediators such as prostaglandins, cytokines (IL-1β, TNF-α, IL-6), LTB 4 and C5a components, leukotrienes (B4 and PGE2) and chemokines (CXCL8) and CCL2). These inflammatory mediators stimulate increased vascular permeability and the migration of leukocytes and neutrophils to the abdominal cavity (Hall et al., 1998). Leukocyte migration has the role of recognizing and neutralizing foreign agents/aggressors, thus being an important step in the development of the inflammatory process (Kumar et al., 2014). The decrease in cell migration indicates the anti-inflammatory activity of new substances (de Jesus et al., 2023; de Lima et al., 2023). The results demonstrate that *P. heyneanus* essential oil did not have an acute anti-inflammatory effect.

So, although PhEO displayed low anti-inflammatory activity, Patchoulol, its major component, is known to inhibit the production of iNOS and IL-6, activate NF-κB via suppressing IκB-α degradation and p65 nuclear translocation, inhibit ERK1/2 activation by suppressing their phosphorylation, and to have anti-inflammatory activity mediated by inhibiting ERK-derived NF-κB activation (Jeong et al., 2013). This fact can be due to the essential oil constituents acting synergistically because their main components when used as references have less or more activity than the essential oil. Therefore, synergism and antagonism studies must be developed (Miguel, 2010).

## 5 Conclusion

*Pogostemon heyneanus* essential oil composition was minimally influenced by climatic parameters over a seasonal period. Furthermore, the species is a source of patchoulol, the major component of its essential oil.

*Pogostemon heyneanus* essential oil does not present acute toxicity, nor were significant effects of central and peripheral analgesia observed. Tests about neurogenic and/or inflammatory pain proved inefficient, indicating that *P. heyneanus* essential oils do not act on certain biological activities despite the plant having broad ethnopharmacological indications. Moreover, the low influence of the climatic parameters on chemical composition can infer that this pharmacological activity is also not subject to climatic variations, that is, it does not change due to the climate.

## Data Availability

The raw data supporting the conclusions of this article will be made available by the authors, without undue reservation.
